# Obstetric Trauma Following Empiric External Cephalic Version by a Traditional Birth Attendant in a Grand Multipara: Intraoperative Discovery of Fetus and Placenta in the Abdominal Cavity

**DOI:** 10.7759/cureus.95687

**Published:** 2025-10-29

**Authors:** Maria Jose Guaman, Jose L Guaman, Cosme Zelaya, Marlene J Rousseau, Mauricio Rousseau Crespo

**Affiliations:** 1 Faculty of Medicine, Universidad Católica Boliviana San Pablo, Santa Cruz de la Sierra, BOL; 2 Department of Obstetrics and Gynecology, Hospital de la Mujer Dr. Percy Boland Rodriguez, Santa Cruz de la Sierra, BOL; 3 Department of Emergency Medicine, Hospital de la Mujer Dr. Percy Boland Rodriguez, Santa Cruz de la Sierra, BOL; 4 Department of Gynecology, Universidad Católica Boliviana San Pablo, Santa Cruz de la Sierra, BOL

**Keywords:** breech baby, cesarean section, emergency obstetric hysterectomy, external cephalic version (ecv), fetal mortality, grand multiparity, maternal morbidity, non-hospital setting, uterine rupture

## Abstract

Cesarean delivery has become one of the most frequent interventions in obstetrics, and abnormal fetal presentation continues to be a major indication. External cephalic version (ECV) is commonly used to reduce the need for cesarean delivery, but when performed in uncontrolled settings, it may lead to serious complications. We report the case of a 39-year-old grand multipara (G11P11) referred from a rural community after undergoing an emperic ECV attempted by a traditional birth attendant eight days before admission. She developed premature rupture of membranes with persistent leakage for one week, followed by severe abdominal pain, intrauterine fetal demise at 34 weeks, sepsis, and severe anemia. Ultrasound revealed oligohydramnios and fetal death. An urgent exploratory laparotomy was performed with antibiotic coverage and transfusion. Intraoperatively, a complete uterine rupture with retroperitoneal extension was identified, with the intact amniotic sac containing the fetus free in the left iliac fossa and the placenta completely separated within the abdominal cavity. Emergency hysterectomy with left oophorectomy, serosal bowel repair, bilateral uterine artery ligation, and extensive abdominal lavage was undertaken. The patient recovered favorably with intensive postoperative care and was discharged on day eight. This case emphasizes that uterine rupture can occur even in women without previous uterine surgery, particularly in the context of extreme multiparity and non-medical obstetric practices. It highlights the importance of performing ECV exclusively in hospital settings, ensuring timely referral, and strengthening community education to prevent catastrophic maternal outcomes.

## Introduction

Cesarean delivery rates have continued to rise worldwide, raising concerns about maternal morbidity and the long-term implications for reproductive health [1]. Among the most frequent indications, abnormal fetal presentation remains a major contributor, often leading to operative delivery despite advances in obstetric care [2]. This trend underscores the ongoing need for strategies that can safely reduce cesarean rates while preserving maternal and neonatal outcomes.

External cephalic version (ECV) is a well-established intervention aimed at reducing cesarean deliveries by converting breech or transverse presentations into cephalic positions [1]. When performed under controlled hospital conditions with proper monitoring, ECV demonstrates favorable success rates of 50-70%, with many successful cases resulting in spontaneous vaginal birth [2,3]. Its broad adoption has made it a valuable component of modern obstetric practice, particularly in settings where cesarean rates are high and efforts are directed toward optimizing vaginal delivery.

Although generally regarded as safe, ECV is not devoid of risk. Transient complications such as fetal heart rate decelerations or maternal discomfort are relatively common, while more significant events like premature rupture of membranes, placental abruption, and fetomaternal hemorrhage are reported in a small percentage of cases [3,4]. Catastrophic outcomes, including uterine rupture, are exceedingly rare and often limited to isolated case reports. Rupture involving intra-abdominal expulsion of the fetus and placenta is particularly devastating and carries high maternal and fetal morbidity [5]. 

Given the scarcity of such reports, documenting these events is essential to strengthen clinical awareness and refine risk stratification in obstetric practice. The present case involves a grand multipara who experienced complete uterine rupture with intra-abdominal expulsion of the fetus and placenta following an empiric ECV performed by a traditional birth attendant. This rare presentation illustrates how unsafe obstetric maneuvers conducted outside hospital supervision can rapidly escalate into life-threatening emergencies, even in women without a prior uterine scar.

## Case presentation

A 39-year-old grand multipara (G11P11, all prior vaginal deliveries with live newborns) was referred to the emergency department from her rural community with obstetric trauma. According to her family, approximately eight days earlier, a local traditional midwife had performed an EVM, after which the patient developed premature rupture of membranes with continuous fluid leakage lasting about seven days.

**Figure 1:**
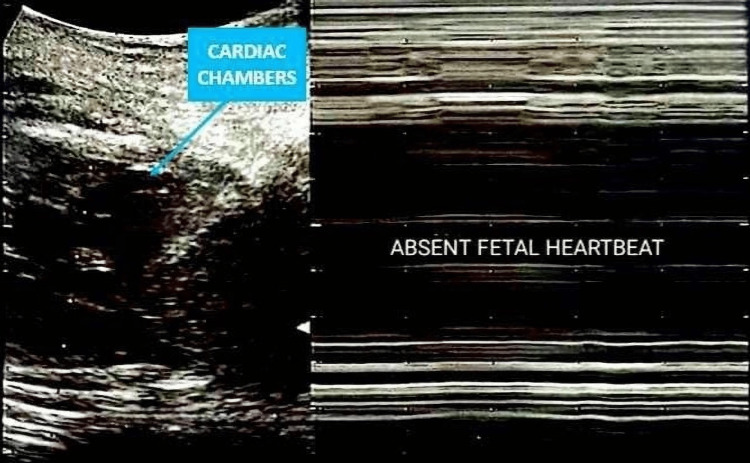
Obstetric ultrasound confirming intrauterine fetal demise Ultrasound image showing the fetal cardiac chambers without detectable activity. The M-mode tracing on the right demonstrates the absence of rhythmic oscillations, consistent with absent fetal heartbeat.

At the time of hospital admission, she presented with severe abdominal pain, intrauterine fetal demise at an estimated gestational age of 34 weeks, and clinical signs of chorioamnionitis and uterine sepsis. Her medical history was remarkable for extreme multiparity without any prior cesarean deliveries. Obstetric ultrasound revealed a posterior right-fundal placenta, severe oligohydramnios with a maximum pocket of less than one centimeter, and confirmed intrauterine fetal demise, as illustrated in Figure 1.

**Figure 2:**
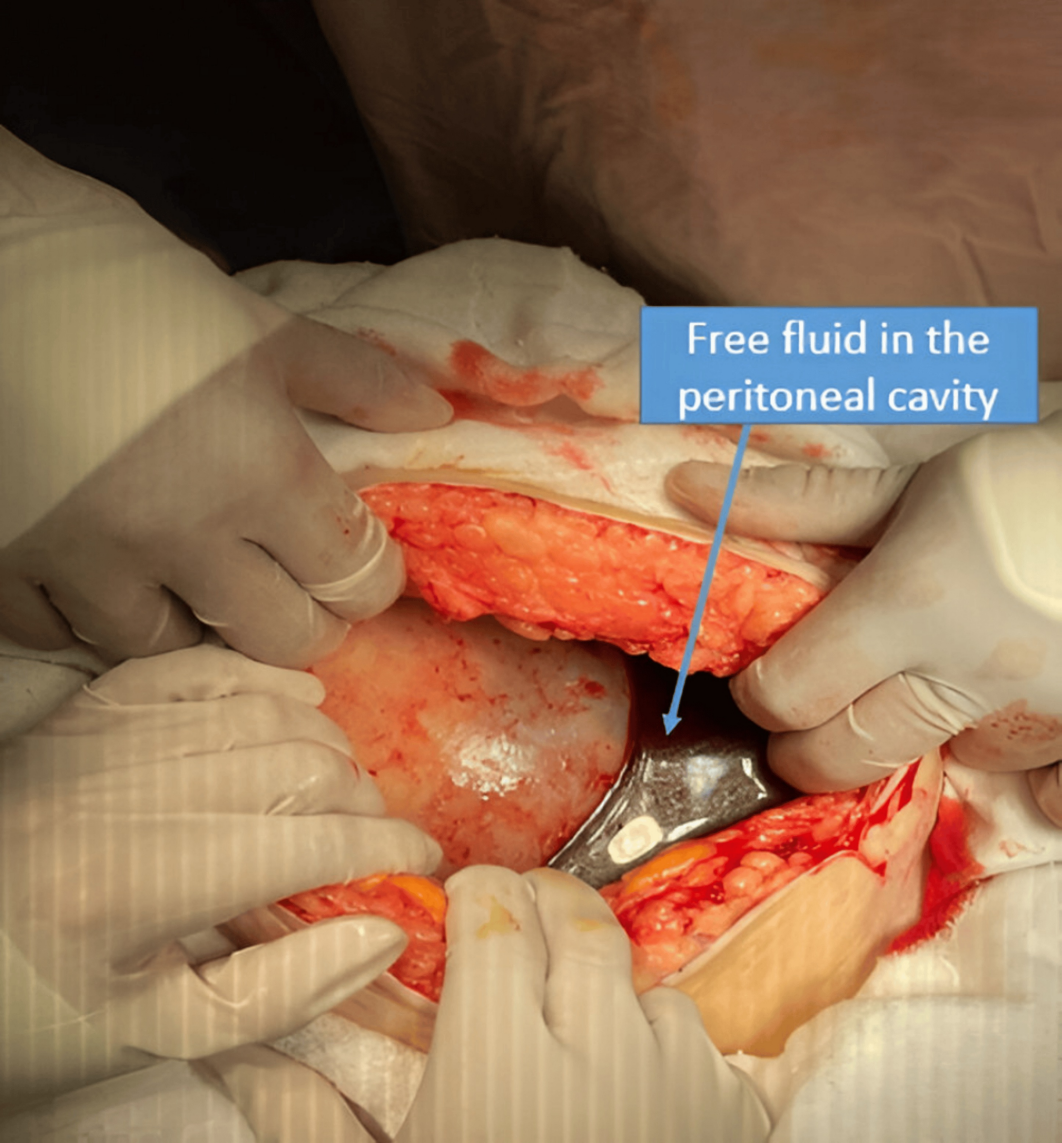
Intraoperative view demonstrating free fluid in the peritoneal cavity Blood clots identified upon entry into the peritoneal cavity during exploratory laparotomy. This finding was associated with complete uterine rupture and intra-abdominal extrusion of the fetus and placenta.

Initial laboratory tests showed hemoglobin of 7 g/dL. An emergency exploratory laparotomy was decided under broad-spectrum antibiotic coverage (metronidazole 500 mg IV and amikacin 1 g IV) and transfusion of one unit of packed red blood cells prior to surgery. A midline infraumbilical incision was performed, and upon entering the peritoneal cavity, a moderate amount of free blood and clots was found (Figure 2).

**Figure 3:**
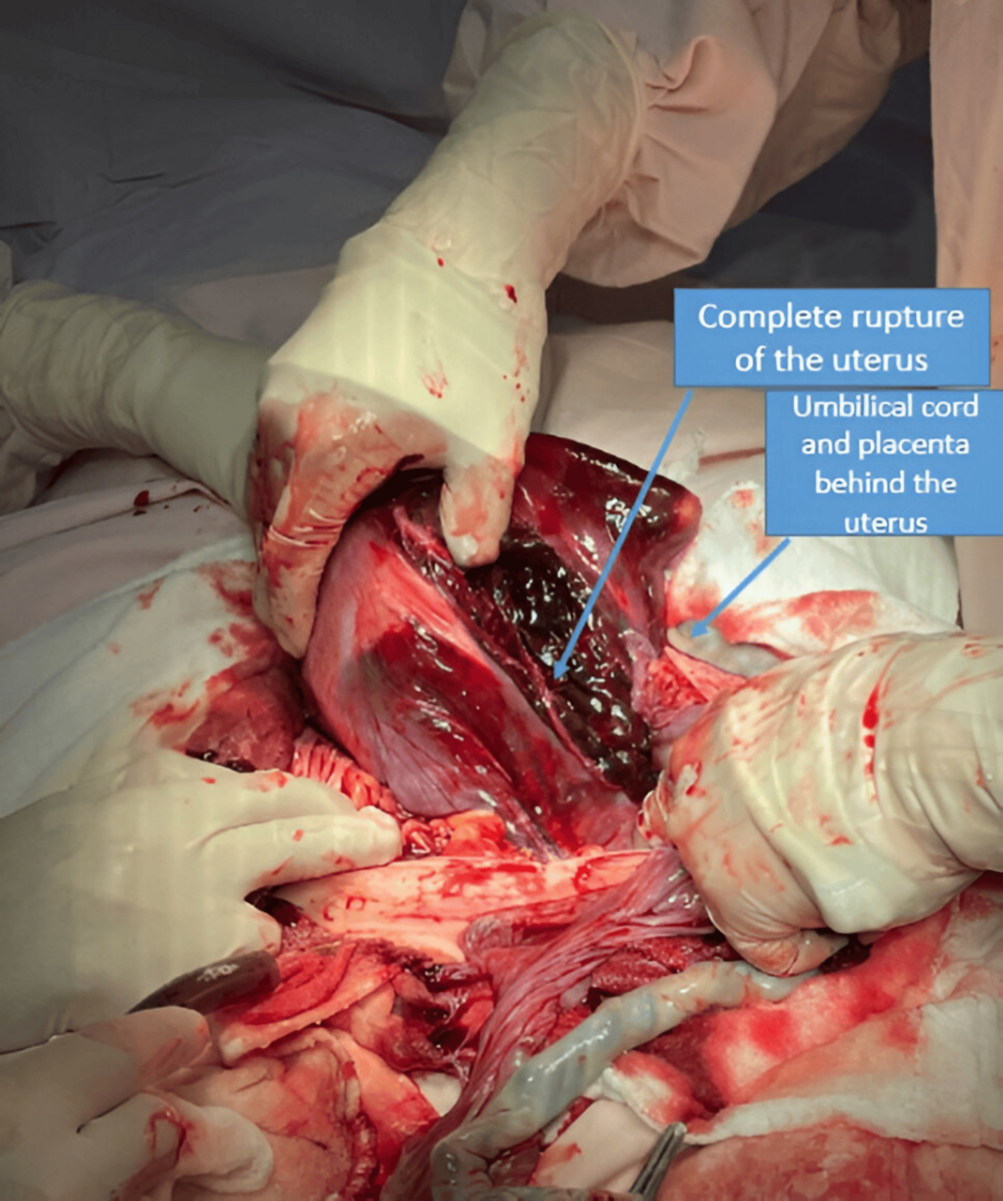
Intraoperative view of complete uterine rupture with placental extrusion Complete rupture of the uterus, extending from the lower uterine segment to the lateral wall, with distortion of the uterine anatomy and active bleeding sites. The placenta is visualized extruded through the rupture, consistent with catastrophic obstetric trauma following an attempted manual external cephalic version.

**Figure 4:**
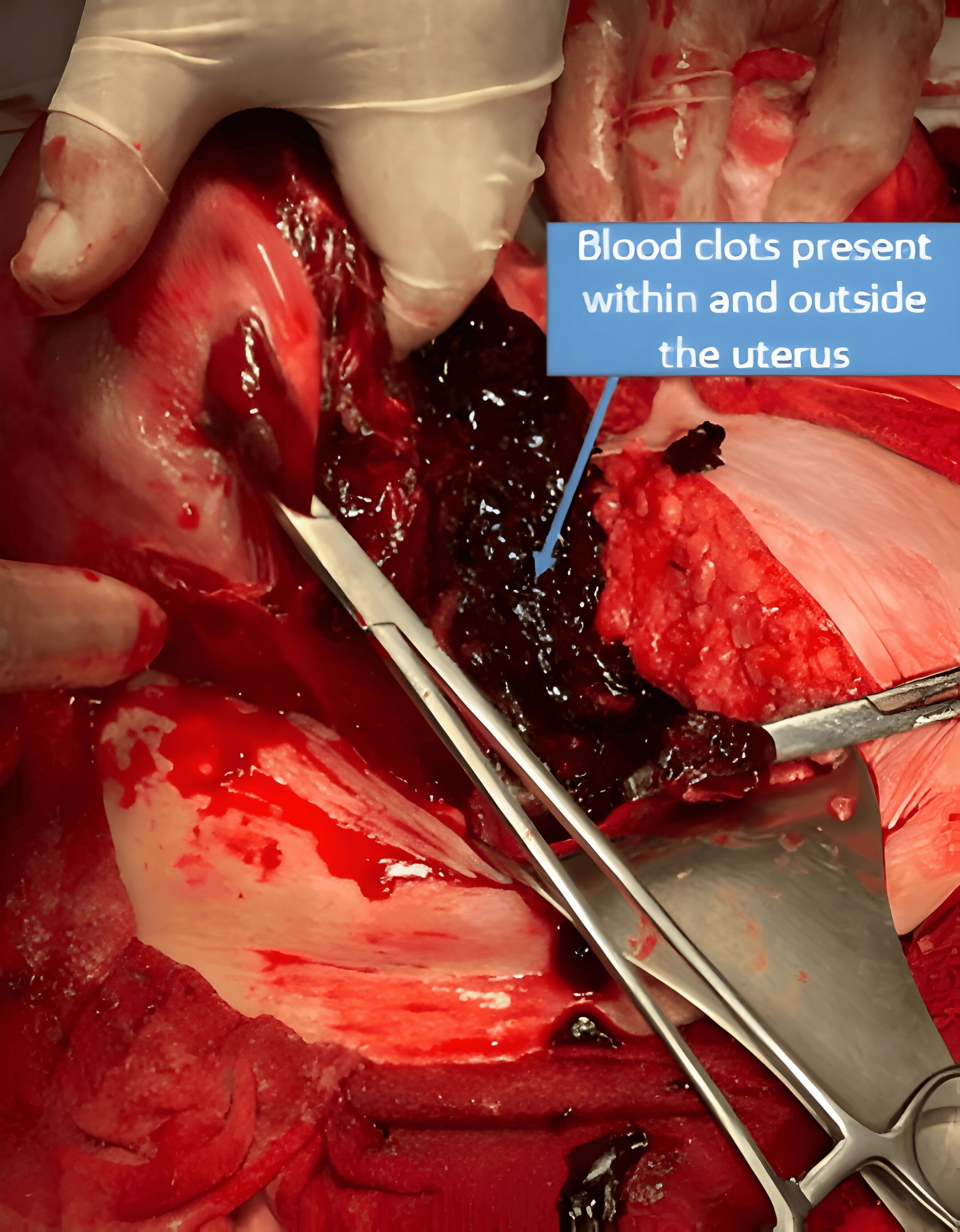
Intraoperative view showing extensive intrauterine and extrauterine blood clots Large accumulation of blood clots both within and outside the uterus. This finding reflects massive hemorrhage associated with complete uterine rupture and intra-abdominal extrusion of the fetus and placenta.

During exploration, the intact amniotic sac containing the fetus was identified, freely located in the left iliac fossa, along with the placenta, completely detached within the abdominal cavity. A complete uterine rupture was confirmed, extending from the lower uterine segment to the left lateral border with retroperitoneal extension, causing total distortion of the uterine anatomy and multiple sites of active bleeding (Figures 3, 4).

**Figure 5:**
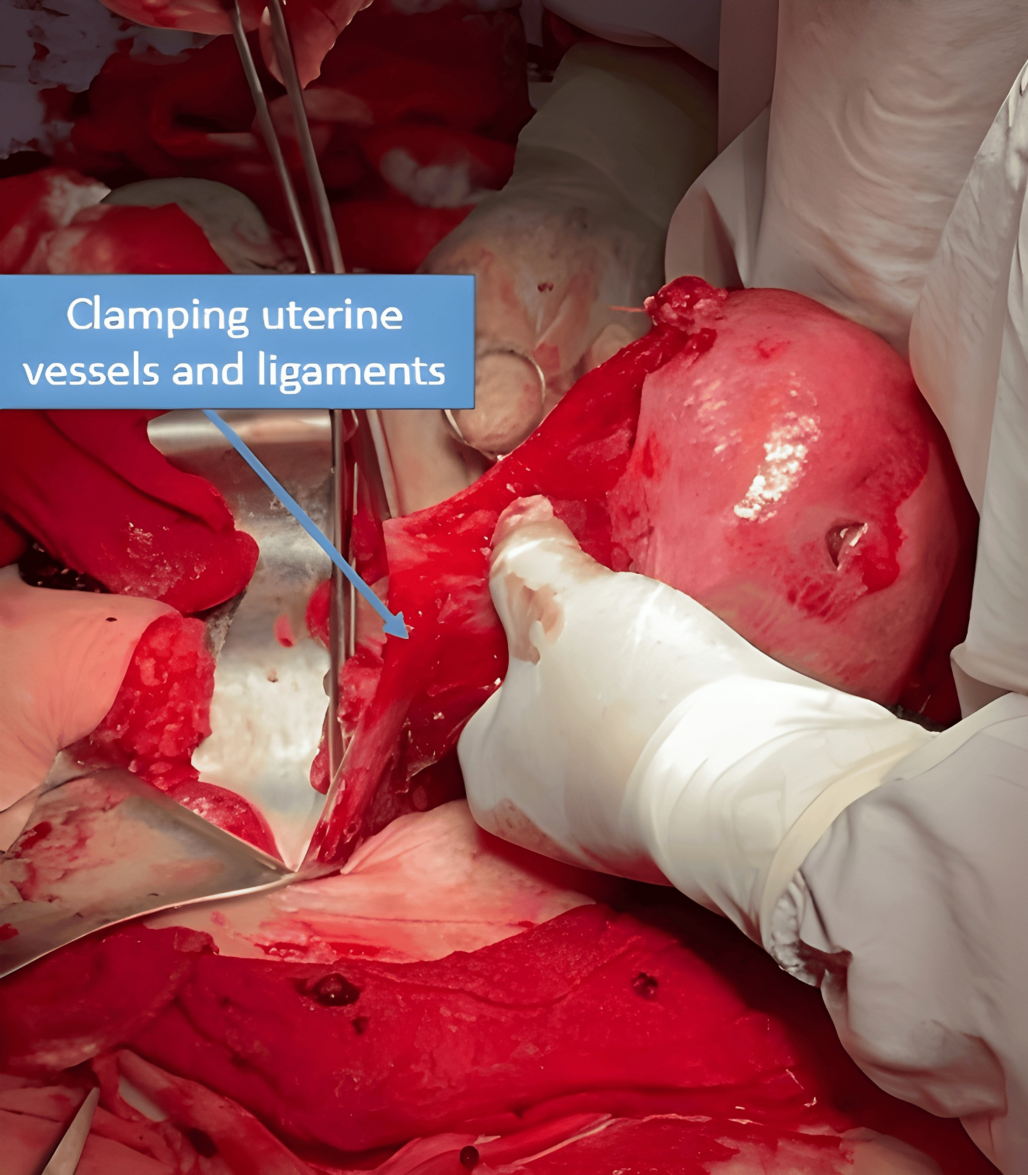
Intraoperative view during clamping of uterine vessels and ligaments for total hysterectomy This maneuver was required due to extensive uterine rupture, uncontrolled hemorrhage, and distortion of the normal anatomy.

Given the extent of the injury and the bleeding, an emergency total hysterectomy was performed as shown in Figure 5. 

**Figure 6:**
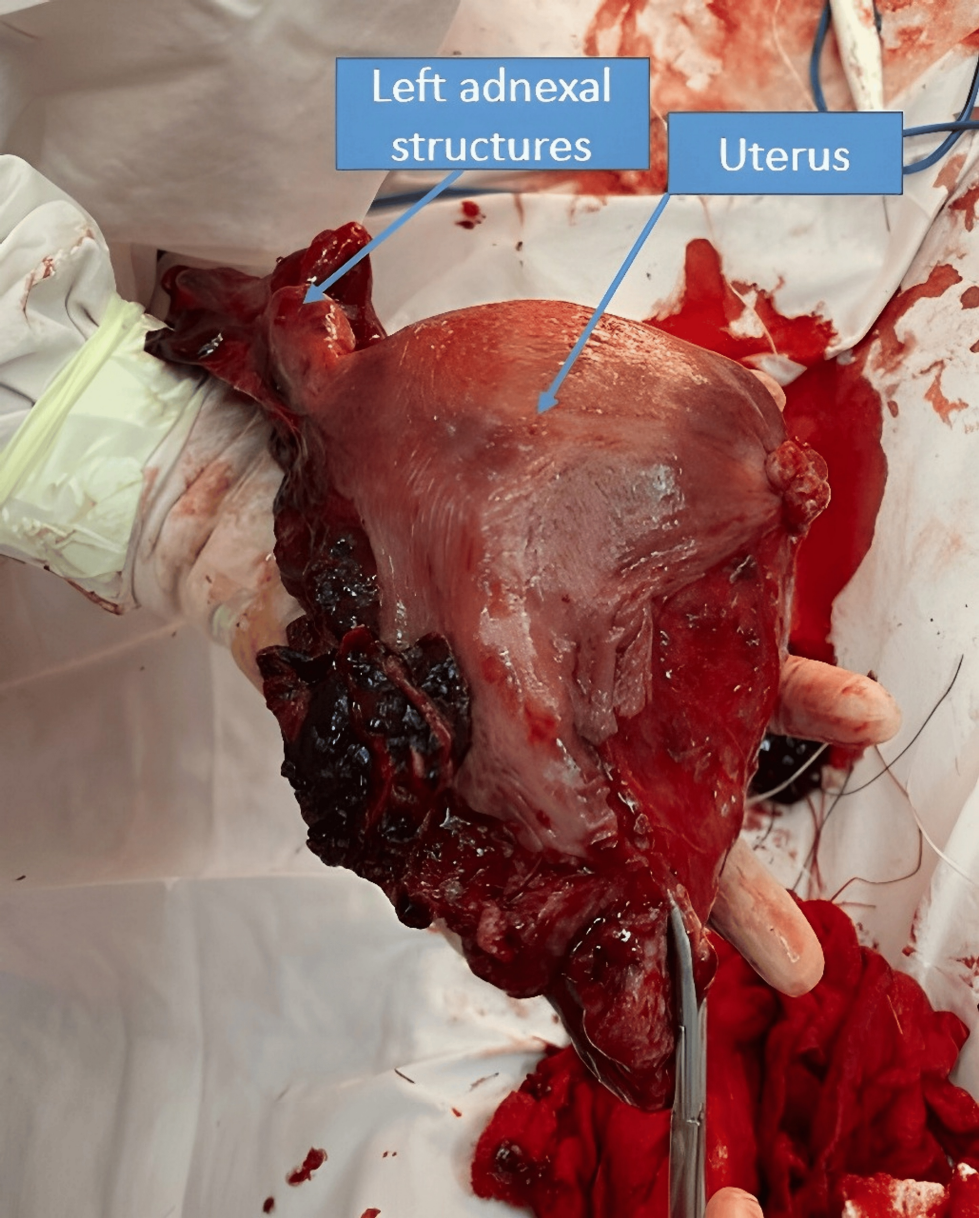
Uterine specimen with left adnexa after emergency total hysterectomy Surgical specimen of the uterus with the left adnexa removed en bloc, showing extensive rupture and distortion of the uterine wall with associated hematomas. The specimen highlights the severity of the uterine disruption that necessitated an emergency total hysterectomy following catastrophic rupture.

**Figure 7:**
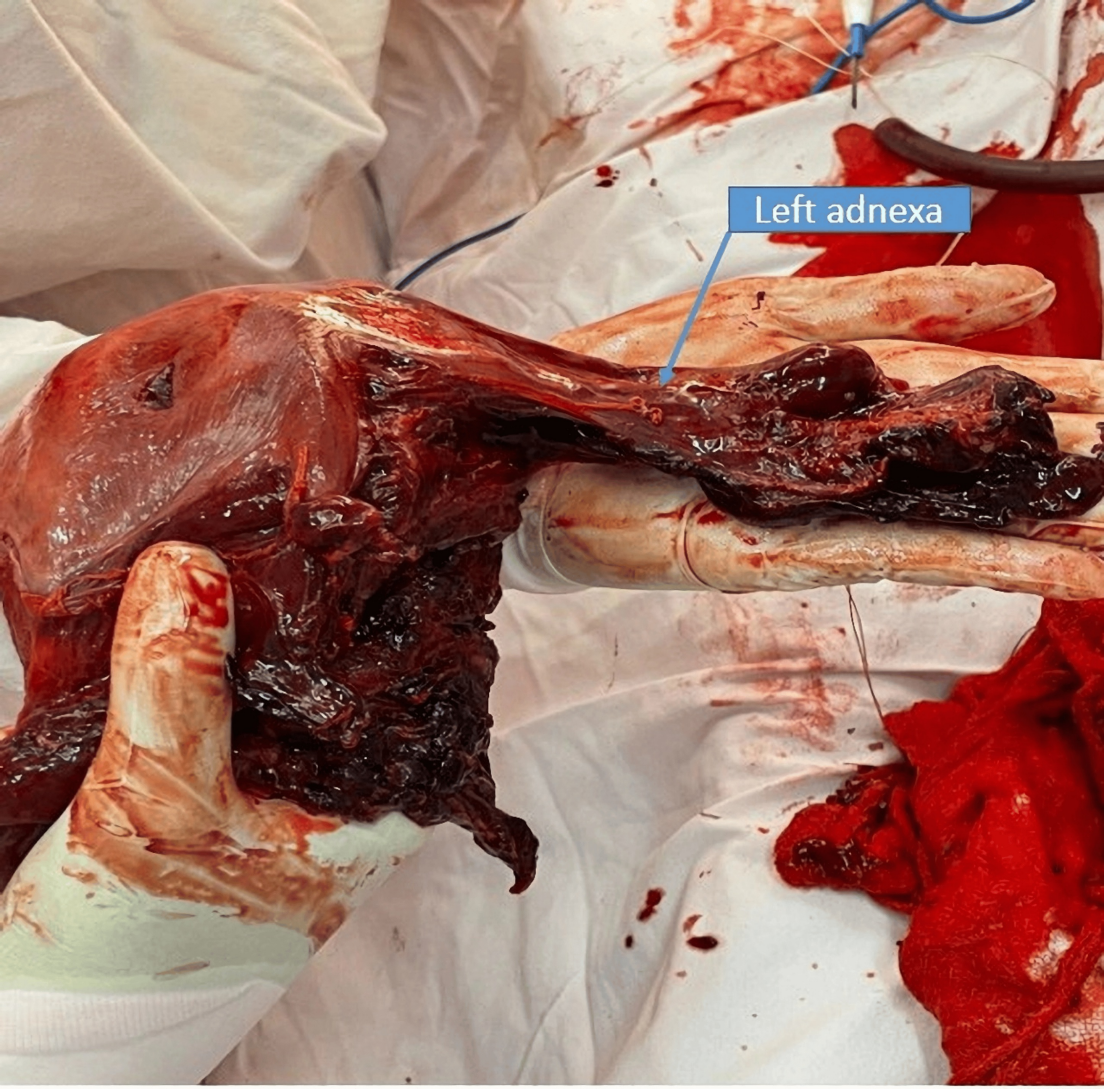
Uterine specimen with left adnexa removed en bloc The image illustrates the extent of uterine rupture and adnexal involvement, necessitating emergency total hysterectomy.

The right ovary was preserved while the left ovary was removed together with the surgical specimen due to complete loss of its anatomical structure (Figures 6, 7). 

**Figure 8:**
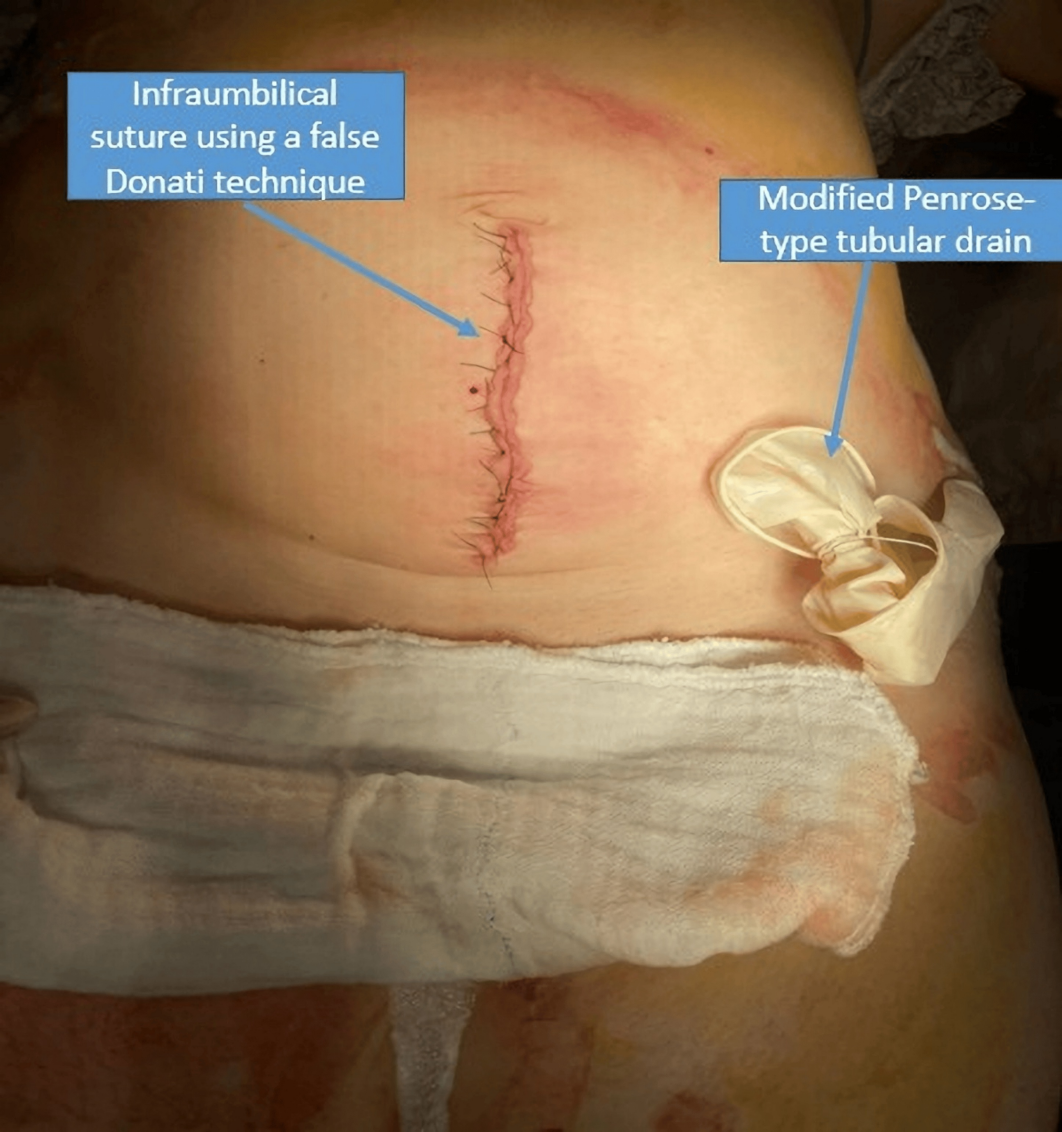
Postoperative abdominal incision with false Donati suture and modified Penrose-type drain Postoperative photograph showing the infraumbilical midline incision closed with a false Donati suture technique. A modified Penrose-type tubular drain is placed in the left flank for postoperative monitoring and fluid evacuation. The image illustrates the surgical closure and drainage strategy following emergency laparotomy and hysterectomy.

Intraoperatively, a retroperitoneal hematoma (zones 2 and 3, left side) and a proximal serosal injury of the large bowel were also identified, prompting the involvement of the general surgery team. Skeletonization and ligation of the uterine arteries were performed, along with repair of the intestinal lesion and thorough abdominal cavity lavage with warm normal saline, leaving a drain in the left flank. The abdominal cavity was subsequently closed, as demonstrated in Figure 8.

The patient received a second intraoperative packed red blood cell transfusion and, subsequently, in the intensive care unit, two additional units, along with completion of the antibiotic regimen and supportive therapy (omeprazole, vitamin K, nifedipine, and ketorolac). Postoperative evolution was favorable, with progressive recovery and no additional infectious complications. The patient was discharged from the hospital eight days later in stable condition.

## Discussion

Uterine rupture in an unscarred uterus is uncommon but carries high maternal morbidity and mortality. Although most cases occur in women with a uterine scar, factors such as extreme multiparity and inappropriate external maneuvers are also determining contributors [6]. In this case, the patient (G11P11) with no prior cesarean deliveries developed a complete rupture after an EVC performed outside the hospital setting.

Recent reports have described similar cases of fetal and placental extrusion into the abdominal cavity, confirming that rupture can be extensive even in intact uteri [7-9]. Other studies highlight that advanced multiparity increases myometrial fragility and may precipitate rupture even in the absence of a previous scar [10]. Our patient matched these risk profiles but differed in presenting a subacute course, with evolution over several days and associated complications such as retroperitoneal hematoma and bowel injury, findings that are rarely described in the literature.

With respect to ECV, the evidence supports its safety when performed under controlled conditions, with fetal monitoring and immediate cesarean capability [11,12]. However, empiric maneuvers in rural areas, as occurred in this case, may generate tangential forces that predispose to uterine tears and intraperitoneal hemorrhage. The observed retroperitoneal hematoma coincides with reports linking obstetric trauma to pelvic vascular injuries [13].

Surgical management depends on the extent of the lesion and maternal stability. Although successful uterine repairs have been reported [9,14], total hysterectomy was the safest option in this case, given the anatomic distortion and multiple bleeding sites. Rapid transfusion support, broad-spectrum antibiotics, and a multidisciplinary approach were decisive for the favorable outcome. This case underscores the need for early diagnosis, aggressive surgical management, and above all, prevention, ensuring that obstetric maneuvers such as ECV are performed exclusively in appropriate hospital settings.

## Conclusions

This case demonstrates that uterine rupture, although uncommon in patients without a prior cesarean history, may occur in the context of extreme multiparity and empiric obstetric maneuvers performed outside the hospital setting. The subacute course with fetal and placental extrusion, along with retroperitoneal extension and associated bowel injury, reflects the potential severity of this condition. Immediate surgical management, multidisciplinary intervention, and transfusion support allowed for a favorable maternal outcome. This case emphasizes the importance of strict hospital protocols for procedures such as ECV, the prevention of empiric practices in rural communities, and early referral as essential strategies to reduce maternal morbidity and mortality.
